# Generation of Human *CRY1* and *CRY2* Knockout Cells Using Duplex CRISPR/Cas9 Technology

**DOI:** 10.3389/fphys.2019.00577

**Published:** 2019-05-09

**Authors:** Teresa Börding, Ashraf N. Abdo, Bert Maier, Christian Gabriel, Achim Kramer

**Affiliations:** ^1^Charité Universitätsmedizin Berlin, Corporate Member of Freie Universität Berlin, Humboldt-Universität zu Berlin, and Berlin Institute of Health, Laboratory of Chronobiology, Berlin, Germany; ^2^Berlin Institute of Health, Berlin, Germany; ^3^Einstein Center for Neurosciences Berlin, Berlin, Germany

**Keywords:** circadian, CRISPR/Cas9, cryptochrome, U-2 OS, duplex

## Abstract

Circadian clocks are endogenous oscillators essential for orchestrating daily rhythms in physiology, metabolism and behavior. While mouse models have been instrumental to elucidate the molecular mechanism of circadian rhythm generation, our knowledge about the molecular makeup of circadian oscillators in humans is still limited. Here, we used duplex CRISPR/Cas9 technology to generate three cellular models for studying human circadian clocks: *CRY1* knockout cells, *CRY2* knockout cells as well as *CRY1*/*CRY2* double knockout cells. Duplex CRISPR/Cas9 technology efficiently removed whole exons of *CRY* genes by using two guide RNAs targeting exon-flanking intron regions of human osteosarcoma cells (U-2 OS). Resulting cell clones did not express CRY proteins and showed short period, low-amplitude rhythms (for *CRY1* knockout), long period rhythms (for *CRY2* knockout) or were arrhythmic (for *CRY1*/*CRY2* double knockout) similar to circadian phenotypes of cells derived from classical knockout mouse models.

## Introduction

The circadian clock is an endogenous, molecular self-sustained oscillator that serves to anticipate daily environmental events (Dibner et al., 2010). The main clock is located in the suprachiasmatic nucleus (SCN) and synchronized to the environment by external signals such as light-dark cycles. In addition to the SCN cells, virtually all other peripheral cells contain a molecular oscillator (Balsalobre et al., 1998; Yoo et al., 2004), which is entrained by the master clock in the SCN (Yamazaki et al., 2000). The circadian oscillator is regulated by interlocked transcription/translation feedback loops. Briefly, the transcriptional activators BMAL1 and CLOCK as a heterodimer activate gene expression of target genes harboring E-boxes in their promotor region (Gekakis et al., 1998). This includes two cryptochrome (*CRY*) genes, and three period (*PER*) genes as well as reverbα and retinoic acid receptor-related orphan receptor-α (*RORα*) (Buhr and Takahashi, 2013). PER and CRY proteins form the core of a large high molecular-weight complex that translocates to the nucleus to inhibit CLOCK/BMAL1 transactivation activity (Aryal et al., 2017). Most of our understanding about the circadian oscillator in mammals is derived from genetic loss-of-function as well as biochemical studies performed in mice. Although in recent years, human cell culture models have been exploited to investigate the circadian clock in humans, true knockout studies have rarely been performed (but see Korge et al., 2015), which precluded definite statements about the role of human clock genes.

The adaptation of CRISPR/Cas (a bacterial defense mechanism against bacteriophages; Doudna and Charpentier, 2014) for mammalian cells facilitates the creation of human gene knockout models. The CRISPR/Cas9 system combines a single guide RNA (sgRNA) with a functional Cas9 inside the targeted cell. The endonuclease Cas9 is targeted to the desired genomic region by the sgRNA and induces a double strand break. The repair of such a break by one of several repair mechanisms often results in random insertions or deletions of base pairs and can lead to frameshift and loss of function of a gene (Rahdar et al., 2015). The duplex CRISPR/Cas9 gene editing strategy (Cong et al., 2013; Ousterout et al., 2015; Savić and Schwank, 2016) aims to delete whole exons by simultaneously introducing double strand breaks in two intron regions flanking exon-intron junctions. By choosing early exons for deletion, whose base pair number is not dividable by three, frame-shifts will be introduced that most likely lead to premature STOP codons and nonsense-mediated RNA decay.

Here, we describe a workflow for the creation of human cell models to study the molecular mechanism of circadian clocks. We used duplex CRISPR/Cas9 technology to knockout two key circadian clock genes, *CRY1* and *CRY2*, in U-2 OS cells, either individually or in combination. The resulting cell lines show the expected genomic alterations, do not express the CRY proteins, and exhibit circadian dynamics similar to primary cells derived from respective mouse knockout models. In summary, we (i) describe an efficient workflow to generate and analyze knockout cells, (ii) make valuable models for circadian rhythms research available as well as, (iii) provide insights into the molecular makeup of the human circadian clock.

## Results and Discussion

**FIGURE 1:**
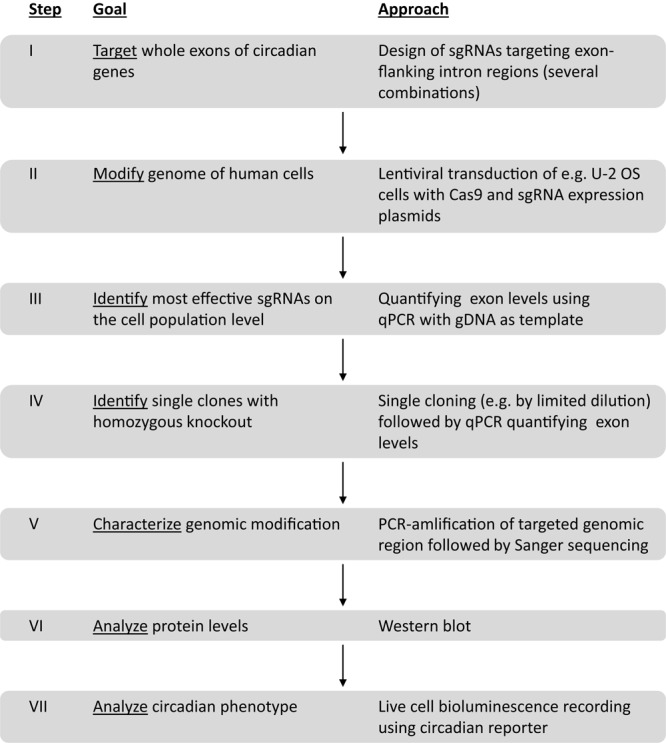
Workflow to create and analyze circadian clock gene knockout cells using duplex CRISPR/Cas9 technology.

To generate and test human cells with targeted deletions of key circadian genes, we worked along the workflow depicted in Figure 1. Important steps in this workflow are: (i) to identify the best sgRNAs for CRISPR/Cas9 gene editing, (ii) to screen for homozygous gene knockout at the single cell level, (iii) to sequence the genome of candidate knockout clones; (iv) to quantify protein expression of candidate knockout clones. We set two specific criteria to be met for a putative knockout clone, before we test its circadian phenotype and correlate it to the genetic perturbation: firstly, the genomic sequence demonstrates a loss of exon on both alleles and secondly, the corresponding protein is undetectable. Any clone that did not meet the above criteria was rejected, and - since the goal of this work was to identify and characterize true positives - not further investigated.

**FIGURE 2:**
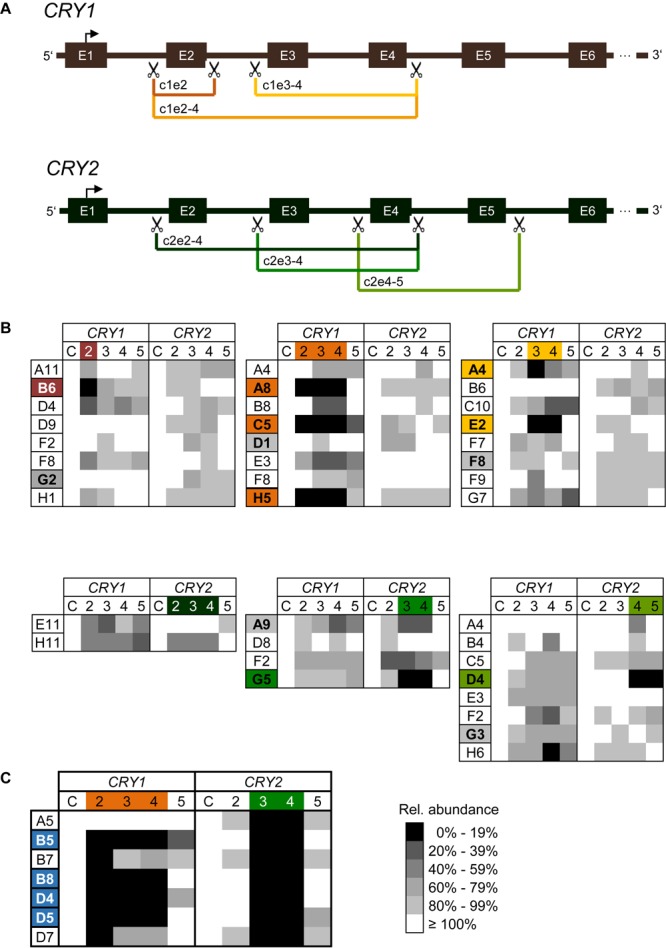
CRISPR/Cas9-mediated generation of *CRY1* and *CRY2* knockout cells **(A)** Schematic overview of CRISPR/Cas9-mediated exon deletion strategy. SgRNAs were designed to introduce a double strand break in intron regions upstream and downstream of target exons (scissors). Deletion of target exons induces a frameshift resulting in a premature STOP codon. For each *CRY* gene three (out of six) different guide RNA combinations are exemplarily shown in different colors. E.g., c1e2-4 refers to a sgRNA combination that aimed at the deletion of exons 2 to 4 of *CRY1*. E: exon. **(B)** Relative genomic abundance of targeted exons in single cell clones. Cell populations as indicated in Supplementary Figure 1 were sub-cloned, and genomic DNA of individual clones was analyzed for exon deletions using qPCR. As control C, abundance of 3′-UTR regions of each gene were quantified. Single cell clones with undetectable levels of targeted exons were maintained for further analysis (marked in the corresponding color), single cell clones with minimal reduction in all screened genomic loci were maintained as controls (marked in gray). **(C)** Generation of *CRY1*/*CRY2* double knockout cells. The *CRY2* knockout cell clone G5 [see **(A)**], was transduced with Cas9 and sgRNA expression vector targeting exons 2–4 of *CRY1*. Single cell clones were analyzed for genomic deletion of target exons as described above. Shown are results for six clones, four of which were selected for further downstream analysis.

To knockout *CRY1* and *CRY2*, we applied a duplex CRISPR/Cas9 gene editing strategy, which aimed to delete one or more exons at the *CRY* genomic loci by a simultaneous Cas9-mediated cleavage at two positions. Target sites were located in intron regions, which flank exons, whose deletions lead to a shift in the reading frame and thus to premature STOP codons. Accordingly, we designed several combinations of sgRNAs (Figure 2A) using the CRISPOR tool that suggests sgRNAs with specific target cleavage sites while minimizing possible off-target effects (Haeussler et al., 2016). The corresponding oligonucleotides were ligated into the lentiCRISPRv2 plasmid (Sanjana et al., 2014), lentiviruses were produced and U-2 OS *Bmal1*-luciferase reporter cells (an established cellular circadian clock model; Maier et al., 2009) were transduced with lentivirus mixtures.

To test which sgRNA combination is most efficient to delete targeted exons, we performed quantitative polymerase chain reaction (qPCR) with genomic DNA of transduced cell populations as a template and primer pairs amplifying the indicated exons. The more efficient the Cas9-mediated deletion of exons occurs, the less abundant the qPCR-generated amplicons are predicted to occur at the target exons, while non-targeted exons should be amplified similar to control genomic regions. Indeed, for most combinations of sgRNAs targeting the various exons in *CRY1* and *CRY2*, we observed a decrease in genomic abundance of the respective exons already in the cell populations by ∼50% and more. In contrast, for non-targeted regions (within the same gene or within the other *CRY* gene) we saw only slight variations in the signal (Supplementary Figure 1). For example, targeting exon 2 of the *CRY1* gene led to an about 40% reduction of exon 2 abundance at the genomic DNA level, while signals for other *CRY1* exons were not reduced and for *CRY2* exons varied only slightly (note, that we do not highlight signals higher than 100%). The variability at non-targeted regions was probably due to experimental noise or systematic error, since the various qPCR assays had slightly different efficiencies and the assay has a low dynamic range due to the fact that in cell populations the overall effect is expected to be lower.

To study the genomic modifications at a clonal cell level, we sub-cloned cells by limited dilution from the three sgRNA pairs showing the highest efficiencies for each *CRY* gene on cell population level (indicated with an arrow and according to color, Supplementary Figure 1). To test for exon deletions at the genomic level, we isolated genomic DNA from 69 sub-clones (50 for *CRY1* and 19 for *CRY2*) and quantified targeted exon regions by qPCR. We identified six and two clones (i.e., about 11% of screened clones) with putative deletions in exons corresponding to each of the two alleles of *CRY1* and *CRY2*, respectively (Figure 2B). For other clones, we observed only reduction of genomic exon abundance of 50% or lower indicating deletion of one allele at most. Again, as discussed above, the qPCR-based analysis of genomic exon abundance did not always result in an unequivocal result (e.g., for clone A4 with targeted exons 3–4 of *CRY1*). To create U-2 OS cells lacking both functional CRY1 and CRY2 proteins, we transduced two putative *CRY2* knockout clones (G5: exons 3–4 targeted; D4: exons 4–5 targeted) with lentiviral mixtures targeting exons 2–4 of *CRY1*. From the resulting cell population, we analyzed 92 clones and identified four putative double *CRY1/CRY2* knockout clones, i.e., the success rate was only about 4% (Figure 2C). This lower rate may be because we could not select for transduced cells, as the cell clones already contained the selection marker from the first round of viral transduction (targeting *CRY2*).

To confirm whether the genomic deletion did occur as predicted from sgRNA target sites, we performed PCR analysis with primers located outside of the putatively deleted genomic region (out-out PCR; Supplementary Figure 2A). For 7 of 12 analyzed clones, the size of the amplicons was as predicted for successful deletion (Supplementary Figure 2B), for 3 clones the results were ambiguous and for two clones, we observed PCR products with unexpected sizes. For example, for clones A4 and E2 with exons 3–4 of *CRY1* targeted, the PCR#2 resulted in products larger than 4 kb (only slightly smaller than the expected size for a wild-type clone) rather than in the expected 546 bp product. This suggests that instead of the intended deletion, other genomic rearrangements (small genomic deletions or inversions) occurred (data not shown), and thus these clones did not meet one of the criteria for a knockout candidate clone. Together, these data indicate that most of the clones identified via genomic qPCR showed the expected genomic alterations, however, a careful and thorough analysis is mandatory to sort out false positives.

**FIGURE 3:**
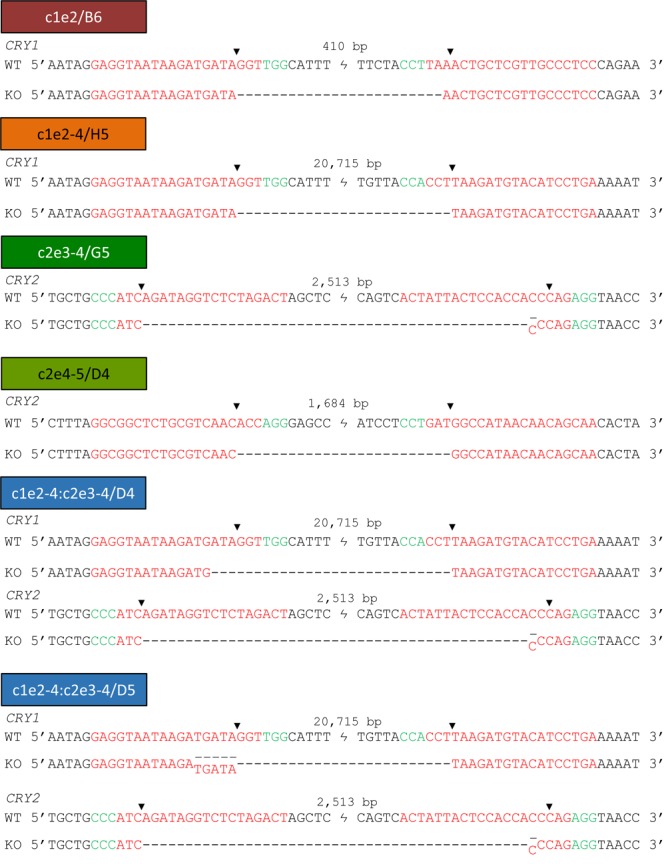
Sequences of single cell clone genomic regions confirm deletion of targeted exons. Out-out-PCR products for each KO clone (see marked bands in Supplementary Figure 2B) were gel-purified, sequenced and aligned to corresponding wild-type sequence. SgRNA recognition sequences are indicated in red, PAM sequence in green and expected Cas9 cutting site by an arrow. Sequence ambiguities suggest that genome editing was not identical for the two alleles.

To study the genomic alterations at the sequence level, PCR products of selected amplifications were sequenced. For most amplicons, we not only observed the expected deletions, but the sequences matched the predicted Cas9 cutting sites, too. In a few cases, we identified two different sequences with predicted deletions indicating slightly different cutting sites for each allele (Figure 3). For example, clone G5 (targeting exons 3–4 of *CRY2*; the “parent” of the double knockout clones) showed two deletion sequences differing by one base. This is an unambiguous demonstration for deletion on both alleles, while a unique sequence indicates a successful deletion on at least one allele, but not necessarily on both.

To study the specificity of the Cas9-mediated genomic alterations, we PCR-amplified and sequenced the genomic regions of the most likely off-target sites (according to the CRISPOR tool) in the genome of clone C5 (with deletion of exons 2–4 of *CRY1*) and clone G5 (targeting exons 3–4 of *CRY2*; the “parent” of the double knockout clones). We analyzed the sequence of the overall most likely off-target sites as well as the most likely site within a protein-coding gene. All off-target sites display at least 4 mismatches compared to the target sites. All sequences showed wild-type sequence indicating absent or very low off-target modifications, at least for the analyzed most likely off-target sites (Table 1).

**Table 1:** Analysis of potential off-target effects of CRISPR/Cas9-mediated genome editing.

			Mismatch	Off-target	% identity with
Target region	Target sequence (sgRNA)	Off-target region	position	score^∗^	wild-type
*CRY1*_post-exon4	TCAGGATGTACATCTTAAGGTGG	intergenic: AC016727.1-RNU6-1145P	....^∗^.......^∗^....^∗∗^	0.4256	Clone C5: 100
*CRY1*_post-exon4	TCAGGATGTACATCTTAAGGTGG	exon: HAND2/HAND2-AS1	.^∗^..^∗∗^.......^∗^......	0.1905	Clone C5: 100
*CRY1*_pre-exon2	GAGGTAATAAGATGATAGGTTGG	exon: KNCN	^∗∗^..^∗^.^∗^.............	0.5647	Clone C5: 100
*CRY1*_pre-exon2	GAGGTAATAAGATGATAGGTTGG	intergenic: DI02-DI02-AS1	^∗^..^∗^......^∗^.......^∗^.	0.5400	Clone C5: 100
*CRY2*_post-exon4	ACTATTACTCCACCACCCAGAGG	intron: TEX26-AS1	^∗^....^∗^.^∗∗^...........	0.7343	Clone G5: 100
*CRY2*_post-exon4	ACTATTACTCCACCACCCAGAGG	exon: PTPRE	......^∗^.^∗∗^.^∗^........	0.2761	Clone G5: 100
*CRY2*_pre-exon3	AGTCTAGAGACCTATCTGATGGG	intergenic: RPll-384P14.1-SNOPvA3	^∗∗^.^∗^........^∗^.......	0.3945	Clone G5: 100
*CRY2*_pre-exon3	AGTCTAGAGACCTATCTGATGGG	exon: PPFIBP1	.....^∗^......^∗^.^∗^..^∗^..	0.0594	Clone G5: 100

*^∗^Given is the CFD off-target score according to Doench et al. (2016).*

**FIGURE 4:**
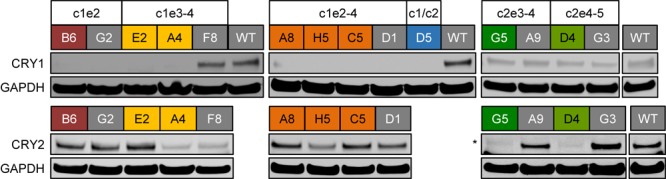
CRY protein levels in CRISPR/Cas9-generated knockout cell clones. Whole cell lysates from indicated single cell clones were analyzed using SDS-PAGE and western blotting. The blots were stained for CRY1 or CRY2 as well as for GAPDH as a loading control. *CRY1*/*CRY2* double knockout clone D5 (blue) was not analyzed for CRY2 expression, since for its “parental” clone, the *CRY2* knockout clone G5 (dark green), we did not detect CRY2 protein. Colored headers indicate the identity of the analyzed clones and their potential genomic deletions. Note, for “gray clones,” we did not detect reduction in relative genomic abundance of target exons (see Figure 2B). ^∗^marks an unspecific band with slightly lower electrophoretic mobility.

If the deletions of exon regions in *CRY1* and *CRY2* result in premature STOP-codons, as predicted, the corresponding proteins should not be produced in their full-length version. To test this, we analyzed the CRY1 and/or CRY2 protein abundance of several identified single clones by western blot using specific antibodies. While we detected CRY1 protein in wild-type clones as well as in *CRY2* deletion clone candidates, CRY1 protein was not detected in clones, which showed deletion of *CRY1* exons on the genomic level (including the *CRY1/CRY2* double knockout clone D5), (Figure 4). Curiously, however, CRY1 was also not detected in some clones, for which we observed no *CRY1* exon deletion by qPCR (G2 and D1) (see Figure 2B). This might either suggest that genomic qPCR gave some false-negative results or, more likely, that in these clones, other genomic alterations occurred (e.g., single cuts with deletions or an inversion of the whole cut region) that could not be detected by qPCR, but prevented antibody recognition or protein production. This demonstrates that also for designated control clones (e.g., transduced clones without deletion), genotyping and phenotyping is of great importance. For all potential *CRY2* knockout clones we did not observe CRY2 protein signals, while CRY2 was detected (with variable intensity probably due to clonal variation) for all control clones as well as for *CRY1* single-knockout clones. Together, these data indicate that the genomic deletions indeed prevented full-length protein expression of the target genes.

**FIGURE 5:**
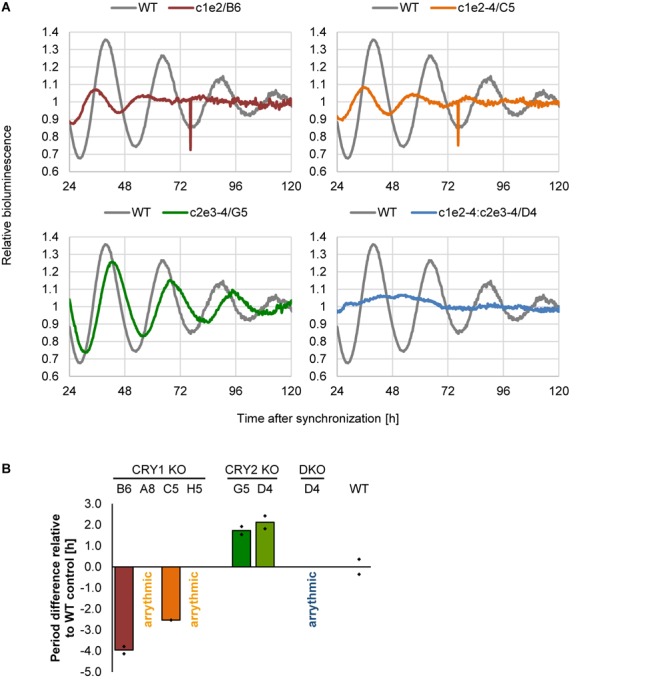
Deletion of CRY proteins alters circadian dynamics. **(A)** Indicated single clones containing a *Bmal1*-luciferase reporter were synchronized with dexamethasone, and bioluminescence rhythms were analyzed for several days. Shown are representative detrended time series. **(B)** Quantification of circadian period for indicated single cell clones (DKO = double knockout). Shown are means of two independent experiments with twelve technical replicas each (clone D5 was measured once with eight technical replicas). Dots indicate the two mean values of the two experiments.

Does deletion of *CRY1* and/or *CRY2* alter circadian rhythms also in human U-2 OS cells? To test this, we synchronized U-2 OS wild-type or *CRY* knockout cells with dexamethasone and measured bioluminescence rhythms for 5–6 days. If CRY proteins have similar roles in the human circadian clock as compared to mouse, *CRY1* knockout should result in short-period, low-amplitude rhythms (or even arrhythmicity, depending on the threshold set for rhythmicity), while *CRY2* knockout should lead to long-period, high-amplitude circadian rhythms (Liu et al., 2007). Double-knockout is predicted to result in clear arrhythmicity (van der Horst et al., 1999). Such results have also been seen in RNA interference studies with human cells (Maier et al., 2009) as well as CRISPR/Cas9-mediated knockout studies with differentiated mouse embryonic stem cells (Tsuchiya et al., 2016). These described phenotypes are exactly what we observed for all of the clones that met the two criteria we set for a true knockout (Figure 5). Together, these phenotypic data showed that loss of CRY proteins in human cells results in circadian phenotypes virtually identical to those of cells from respective classical knockout mouse models, indicating that the molecular makeup of the circadian oscillator is similar between mice and humans.

In conclusion, we describe an efficient workflow for generating and testing human cells with targeted deletions of key circadian genes using duplex CRISPR/Cas9 technology. We created three new cell models to study the circadian clock in humans on a molecular level. Although simplex sgRNA-mediated genomic alteration (using only one sgRNA) showed a very efficient Cas9-mediated cutting in U-2 OS cells (Korge et al., 2015), the duplex sgRNA approach allowed for a better screening of positive clones, since (i) genomic qPCR is more sensitive and robust compared to T7 endonuclease assay used to detect repair-associated insertions or deletions (indels), (ii) successful Cas9-mediated exon deletions always lead to premature STOP-codons, while indels (at least theoretically) create frame shifts in only two thirds of the cases, (iii) the extent of deletion is more predictable, since in most cases no indels occurred upon repair.

## Materials and Methods

### Plasmids and Oligonucleotides

Oligonucleotides specific for the target sites (guide RNAs, Supplementary Table 1) were designed using the CRISPOR tool (Haeussler et al., 2016^1^) and ligated into the lentiCRISPR v2 plasmid (Addgene #52961) (Sanjana et al., 2014) using a BsmBI restriction site.

### Lentivirus Production and Transduction

Lentiviruses were produced in HEK293T cells as described previously (Maier et al., 2009) and virus-containing supernatants were filtered. 3 × 10^5^ U-2 OS *Bmal1*-luciferase reporter cells per well were seeded into 6-well plates in a total volume of 200 μl. Cells were transduced with two lentiviruses (1 ml each, corresponding to the two target sites) supplemented with 0.8 mg/ml protamine sulfate. For single knockout experiments cells were selected for CRISPR/Cas9 positive cells using 10 μg/ml puromycin after 24 h.

### Quantitative Polymerase Chain Reaction (PCR)

Genomic DNA from confluent cells (minimum 48-well format) was isolated using DirectPCR Lysis Reagent (Cell) (Viagen, Los Angeles, CA) and used as template for subsequent PCR. Quantitative PCR was performed with specific primers for each locus (Supplementary Table 2) and the CFX96 C1000 Touch qPCR thermo-cycler (Bio-Rad, Munich, Germany). Obtained data were first normalized to corresponding data from U-2 OS wild-type controls (to adjust for variation in input) and then normalized to the genomic abundance to data from an untargeted control region (3′-untranslated regions of each gene).

### Out-Out PCR and Sequencing

Forward (fw) and reverse (rv) primers were designed to anneal closely 5′ and 3′ of the targeted exons (Supplementary Table 3). The PCR was performed using Phusion High-Fidelity DNA Polymerase. PCR products were gel-purified and Sanger-sequenced using the same primers.

### SDS-PAGE and Western Blot

Western blotting was performed essentially as described in Maier et al. (2009). Briefly, cells were harvested in RIPA lysis buffer containing protease inhibitor cocktail (1:100, Sigma, Japan). Equal amounts of protein were separated by SDS-PAGE using 4–12% Bis-Tris gels (Invitrogen, United States), transferred to nitrocellulose membrane and incubated overnight at 4°C with anti-CRY1 (1:400, Bethyl Laboratories, A302-614A), anti-CRY2 (1:500, Bethyl Laboratories, A302-615A) or anti-GAPDH antibody (1:1000, Santa Cruz, sc-32233). Membranes were probed with HRP-conjugated secondary antibodies (donkey anti-rabbit, Santa Cruz, sc-2305, 1:1000 or goat anti-mouse, Santa Cruz, sc-2005, 1:1000 in TBST) for 1–2 h at room temperature. Detection was performed using the chemiluminescence assay with Super Signal West Pico substrate (Pierce).

### Bioluminescence Recordings

Live cell bioluminescence recordings were performed essentially as described (Maier et al., 2009). Briefly, cells were synchronized using 1 μM dexamethasone for 30 min, washed and cultured in phenol-red-free medium, supplemented with 10 % fetal calf serum, antibiotics and 250 μM D-luciferin (PJK) and placed in a 96-well plate luminometer (Topcount, Perkin Elmer). Bioluminescence recordings were continuously monitored for several days. Time series were analyzed with the ChronoStar software (Maier et al., 2019). Circadian parameters such as period and amplitude were calculated based on the data from 12 to 120 h. The reference time for the determination of the amplitude was 24 h. Rhythmicity of cells was assessed by visual inspection and the goodness of the fit parameter of ChronoStar.

## Author Contributions

AK, BM, and CG conceptualized and designed the study. TB and AA acquired the data. AK, BM, CG, TB, and AA analyzed and interpreted the data and critically revised the manuscript. TB and AK drafted the manuscript.

## Conflict of Interest Statement

The authors declare that the research was conducted in the absence of any commercial or financial relationships that could be construed as a potential conflict of interest.

## Footnotes

1 http://crispor.tefor.net/
