# Supplementary material for: Generation of Human CRY1 and CRY2 Knockout Cells Using Duplex CRISPR/Cas9 Technology
Source: Front Physiol. 2019 May 9;10:577. doi: 10.3389/fphys.2019.00577 (PMC6521593; doi:10.3389/fphys.2019.00577)
Supplement: Supplementary file 1 [file Data_Sheet_1.PDF]

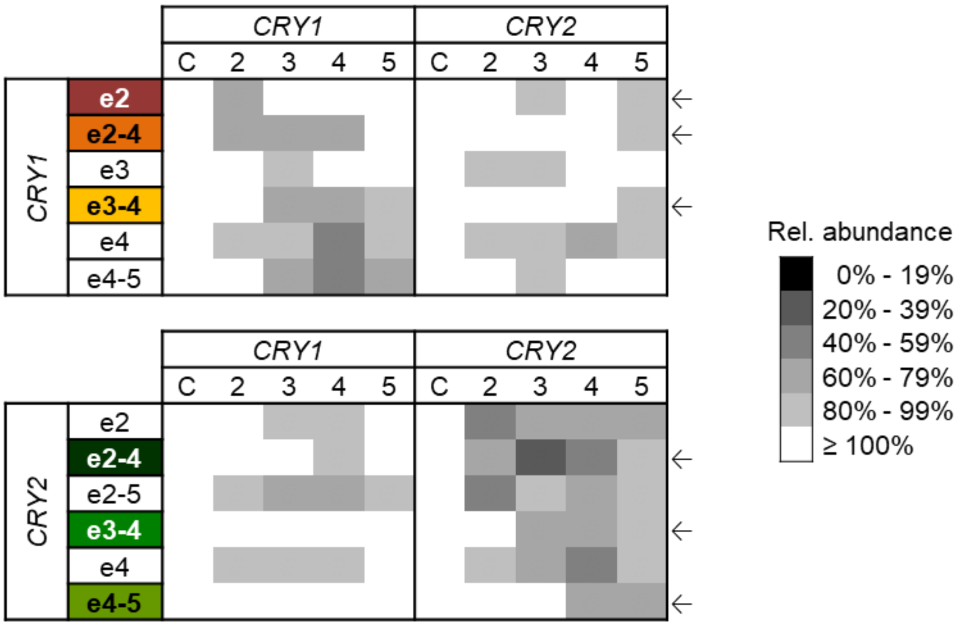

Supplementary Figure 1. CRISPR/Cas9-mediated generation of *CRY1* and *CRY2* knockout cells. Relative genomic abundance of targeted exons in the genomic DNA of U-2 OS cell populations after lentiviral transduction with Cas9 and sgRNA expression vectors, as determined with quantitative PCR. Genomic abundance of targeted exons (exons 2 to 5 for both *CRY* genes) or 3'-UTR as control (C, which was set to 100%) was determined for each target approach. Top panel: six target approaches for *CRY1*; lower panel: six target approaches for *CRY2*. Colors refer to target strategy shown in **Figure 2A**. Arrows indicate those cell populations that were selected for single clone analysis (see **Figure 2B**).

A

#	target exons PCR		Expected PCR product size for WT and KO clones [bp]						
	gene	exon	WT	c1e2	c1e2-4	c1e3-4	c2e3-4	c2e4-5	c1e2-4 c2e3-4
1	*CRY1*	2	1678	1246	-	1678	1678	1678	-
2	*CRY1*	3-4	4430	4430	-	546	4430	4430	-
3	*CRY2*	3-4	3946	3946	3946	3946	1389	-	1389
4	*CRY2*	4-5	2547	2547	2547	2547	-	841	-
5	*CRY2*	4	1227	1227	1227	1227	-	-	-
6	*CRY1*	2-4	>10 kb	>10 kb	1013	>10 kb	>10 kb	>10 kb	1013

B

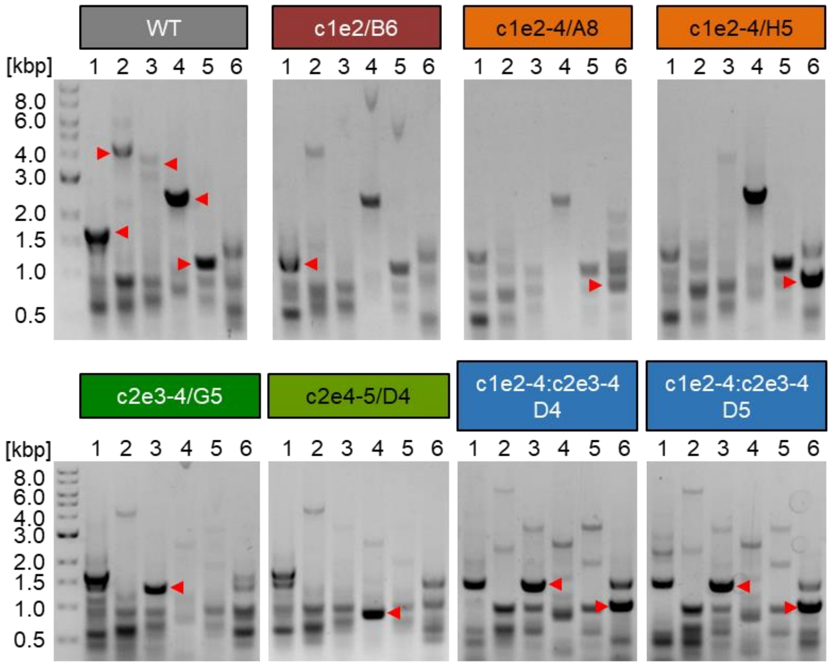

Supplementary Figure 2. Out-out PCR to study genomic alterations of *CRY* knockout single clones. (**A**) Design of the out-out-PCR. To confirm exon deletions in single cell clones, six different PCR experiments were designed to amplify the targeted genomic regions. PCR product size depended on the specific genome editing. The expected sizes of amplicons of knockout - but not wild-type (WT) - clones are marked in grey. (**B**) Out-out PCR products analyzed using agarose gel electrophoresis. Six different primer pairs were used (PCR 1-6; see panel A) to amplify the various targeted genomic loci. Bands specific for the genomic composition of each clone are marked with an arrow. Deletion-specific bands were excised from the gel and sequenced (see **Figure 3**). Colored headers indicate the identity of the analyzed clones and their potential genomic deletions.

Supplementary Table 1. SgRNA sequences

Name	Sequence (5' → 3')*	Genomic target
gR_c1i1-2#1_fw<br>gR_c1i1-2#1_rv	caccgGAGGTAATAAGATGATAGGT<br>aaacACCTATCATCTTATTACCTCc	*CRY1* 5' of Exon 2
gR_c1i2-3#1_fw<br>gR_c1i2-3#1_rv	caccgGGAGGGCAACGAGCAGTTTA<br>aaacTAAACTGCTCGTTGCCCTCCc	*CRY1* 3' of Exon 2
gR_c1i2-3#2_fw<br>gR_c1i2-3#2_rv	caccgTGCATAACCCATTAGATTCT<br>aaacAGAATCTAATGGGTTATGCAc	*CRY1* 5' of Exon 3
gR_c1i3-4#1_fw<br>gR_c1i3-4#1_rv	caccgGCTGCTATTGACCCAATGAC<br>aaacGTCATTGGGTCAATAGCAGCc	*CRY1* 3' of Exon 3
gR_c1i3-4#2_fw<br>gR_c1i3-4#2_rv	caccgTTGGCTAATAAGCTCCCAAA<br>aaacTTTGGGAGCTTATTAGCCAAc	*CRY1* 5' of Exon 4
gR_c1i4-5#1_fw<br>gR_c1i4-5#1_rv	caccgTCAGGATGTACATCTTAAGG<br>aaacCCTTAAGATGTACATCCTGAc	*CRY1* 3' of Exon 4
gR_c1i5-6#1_fw<br>gR_c1i5-6#1_rv	caccgGGTAGTGCGGTAGTAGCTAA<br>aaacTTAGCTACTACCGCACTACCc	*CRY1* 3' of Exon 5
gR_c2i1-2#1_fw<br>gR_c2i1-2#1_rv	caccgCTCCAGCCCTAGTGATCATG<br>aaacCATGATCACTAGGGCTGGAGc	*CRY2* 5' of Exon 2
gR_c2i2-3#1_fw<br>gR_c2i2-3#1_rv	caccgGACTTACCACATCCTATGTC<br>aaacGACATAGGATGTGGTAAGTCc	*CRY2* 3' of Exon 2
gR_c2i2-3#2_fw<br>gR_c2i2-3#2_rv	caccgAGTCTAGAGACCTATCTGAT<br>aaacATCAGATAGGTCTCTAGACTc	*CRY2* 5' of Exon 3
gR_c2i3-4#2_fw<br>gR_c2i3-4#2_rv	caccgGGCGGCTCTGCGTCAACACC<br>aaacGGTGTTGACGCAGAGCCGCCc	*CRY2* 5' of Exon 4
gR_c2i4-5#1_fw<br>gR_c2i4-5#1_rv	caccgACTATTACTCCACCACCCAG<br>aaacCTGGGTGGTGGAGTAATAGTc	*CRY2* 3' of Exon 4
gR_c2i5-6#1_fw<br>gR_c2i5-6#1_rv	caccgTTGCTGTTGTTATGGCCATC<br>aaacGATGGCCATAACAACAGCAAc	*CRY2* 3' of Exon 5

* capital letters correspond to sgRNA sequence

Supplementary Table 2. Primer sequences for qPCR

Name	Sequence (5' → 3')	Genomic target
cry1_e2#1_fw	TTGCTTCAGTGTCTTGAGGATCT	*CRY1* Exon 2
cry1_e2#1_rv	TCTGCTGGTTGTCCACGAAT	
cry1_e3#1_fw	TTGGTGTACATTGTTCCCCTTTC	*CRY1* Exon 3
cry1_e3#1_rv	AATAGCTGCGTCTCGTTCCT	
cry1_e4#1_fw	ACTCAATGGTGGACAACCGC	*CRY1* Exon 4
cry1_e4#1_rv	TCTCTAGTGGTTCCATTTTGCTGA	
cry1_e5#1_fw	TTGTTCTCCCAGTTATTGGGGTTA	*CRY1* Exon 5
cry1_e5#1_rv	GGCCACACTGCAGAGGATAAG	
qCRY1_2f	CCAAATTGGTATTGTAGCCTCTGT	*CRY1* 3' UTR
qCRY1_2r	TGATACTTCCACAAGTTTGGGTCT	
cry2_e2#1_fw	TGGACTCCACAGGTTCCTACT	*CRY2* Exon 2
cry2_e2#1_rv	GTCCCCGGACTACAAACAGG	
cry2_e3#1_fw	GGACAGGTAAGAGATGGGGC	*CRY2* Exon 3
cry2_e3#1_rv	CTCAGCAGGGGCCCAAATTA	
cry2_e4#1_fw	GCTTTCAGGCCATCATCAGC	*CRY2* Exon 4
cry2_e4#1_rv	CGTAGGTCTCGTCGTGGTTC	
cry2_e5#1_fw	TAACCCCCAGTAGCACCGAG	*CRY2* Exon 5
cry2_e5#1_rv	CGGCACGGCTGTTCTGTTAT	
qCRY2_2f	TCCCAGTAGCTGAGCTCCAA	*CRY2* 3' UTR
qCRY2_2r	TGCTCCTGGAAGGAATGGTG	

Supplementary Table 3. Primer sequences for out-out PCR

Name	Sequence (5’ → 3’)	Genomic target
Cry1 Exon 2 fw	AAGCAGTCTTCTGGTACTGCCCCA	*CRY1* 5’ of Exon 2
Cry1 Exon 2 rv	GCACTACAAATTCTGTATTATTGGTAAAA TGTCC	*CRY1* 3’ of Exon 2
Cry1 Exon 3 fw	CTGTGAACTCTGGCAGGTCCCTAA	*CRY1* 5’ of Exon 3
Cry1 Exon 4 rv	AATGGCAGTTTGGAAATACAAGCATAGC	*CRY1* 3’ of Exon 4
Cry2 Exon 3 fw	GCCTCCCTTCTCCCCTCGACA	*CRY2* 5’ of Exon 3
Cry2 Exon 4 fw	AGCCTCCGTTCTCTTCTTCGCC	*CRY2* 5’ of Exon 4
Cry1 Exon 4 rv	CTGAGAAGCCCATGGACACCAGC	*CRY2* 3’ of Exon 4
Cry1 Exon 5 rv	TAGGGGGAACCTGCCCACAGAT	*CRY2* 3’ of Exon 5
